# Chemical Profiling, Bioactivity Evaluation and the Discovery of a Novel Biopigment Produced by *Penicillium purpurogenum* CBS 113139

**DOI:** 10.3390/molecules27010069

**Published:** 2021-12-23

**Authors:** Antonis D. Tsiailanis, Chrysanthi Pateraki, Mary Kyriazou, Christos M. Chatzigiannis, Maria Chatziathanasiadou, Nikolaos Parisis, Ioanna Mandala, Andreas G. Tzakos, Apostolis Koutinas

**Affiliations:** 1 Section of Organic Chemistry and Biochemistry, Department of Chemistry, University of Ioannina, 451 10 Ioannina, Greece; antonis.tsiailanis@gmail.com (A.D.T.); cmchatzigiannis@gmail.com (C.M.C.); m.chatziathanasiadou@gmail.com (M.C.); nikparis@gmail.com (N.P.); 2 Department of Food Science and Human Nutrition, Agricultural University of Athens, 118 55 Athens, Greece; paterakichr@aua.gr (C.P.); meri.kiriazou@outlook.com.gr (M.K.); imandala@aua.gr (I.M.); 3 Biomedical and Analytical Center (BAC), Department of Chemistry, University of Ioannina, 451 10 Ioannina, Greece; 4 Institute of Materials Science and Computing, University Research Center of Ioannina (URCI), 451 10 Ioannina, Greece

**Keywords:** *Penicillium purpurogenum*, biobased colorants, NMR, HRMS, pH stability, cytotoxicity

## Abstract

Biobased pigments are environmentally friendly alternatives to synthetic variants with an increased market demand. Production of pigments via fermentation is a promising process, yet optimization of the production yield and rate is crucial. Herein, we evaluated the potential of *Penicillium purpurogenum* to produce biobased pigments. Optimum sugar concentration was 30 g/L and optimum C:N ratio was 36:1 resulting in the production of 4.1–4.5 AU (namely Pigment Complex A). Supplementation with ammonium nitrate resulted in the production of 4.1–4.9 AU (namely Pigment Complex B). Pigments showed excellent pH stability. The major biopigments in Pigment Complex A were *N-threonyl-rubropunctamin* or the acid form of *PP-R* (red pigment), *N-GABA-PP-V* (violet pigment), *PP-O* (orange pigment) and *monascorubrin*. In Pigment Complex B, a novel biopigment annotated as *N-GLA-PP-V* was identified. Its basic structure contains a polyketide azaphilone with the same carboxyl-monascorubramine base structure as *PP-V* (violet pigment) and *γ-carboxyglutamic acid (GLA)*. The pigments were not cytotoxic up to 250 μg/mL.

## 1. Introduction

Natural pigments are nowadays preferred in the food industry over artificial pigments that are associated with hazardous health effects. Natural pigments are either extracted from plants or are synthesized via microbial fermentation from bacteria, yeast, fungi and algae [1]. Pigment production via microbial fermentation can reach high yields, can be produced from renewable resources and can become a sustainable source of natural pigments since the fermentation process does not compete with farm landing. However, one major bottleneck in the utilization of natural pigments in the food industry is their low stability compared to synthetic pigments [2].

Synthetic pigments are widely used in the food industry, and the safety of certain synthetic pigments has gained the public’s attention. Thus, an interest in natural pigment utilization has been drawing attention [3]. Interest in the commercialization of biobased colorants is increasing in Western countries, yet there are significant concerns regarding the safety of these pigments. Consumption of pigments from *Talaromyces purpureogenus* from rats showed that the complex of pigments that contained purpuride, PP-O, PP-R, pentalsamonin, puractin-A, arginine-monascorubrin, purpurquinone-A, ankaflavin, purpactin-C are safe for consumption [3]. Except from safety, the stability of the pigments is a major challenge in the food industry. Shelf life, temperature and the pH of the food product are the most important factors that could alter the color intensity and may reduce the functional properties of the pigment additives [4]. Thermal stability of red pigments produced by *Penicillium purpurogenum* GH2 was sufficient at pH 6 and up to 80 °C, but a significant decrease in the color intensity was observed when pH slightly dropped [4].

Biobased colorants from filamentous fungi are secondary metabolites and can be categorized into different types of pigmented molecules (e.g., azaphilones, carotenoids, anthraquinones, flavins and others). The necessity to switch from petroleum-based products to natural products is expected to increase biobased colorant production. Food grade colorants are already being produced industrially via fermentation. Natural Red™ is produced by *Penicillium oxalicum*, riboflavin is produced by *Ashbya gossypii*, lycopene and β-carotene by *Blakeslea trispora* [5]. *Monascus purpureous* is a fungus widely used in a variety of commercial food products (i.e., fermented mold rice, red koji and Anka) in Asian countries. It can produce yellow (monascin and ankaflavin), orange (rubropunctatin and monascorubin), and red (rubropunctamine and monascorubramine) pigments, while citrinin (a hepato-neuro-toxic mycotoxin) can be a fermentation byproduct [6]. New fungal strains that belong to *Penicillium* species cannot produce mycotoxins, yet they are able to produce *Monascus*-like pigments [7]. These pigments are polyketides and belong to the family of anthraquinones and azaphilones [8].

Industrial application of a biobased product requires the optimization of fermentation conditions to achieve high product concentration and production rates. Initial sugar concentration and carbon to nitrogen ratio are essential factors for secondary metabolites production [9]. Sufficient biomass needs to be produced to maximize pigment production while biomass and pigments compete for the carbon source. Optimum C:N ratio will lead to the production of sufficient biomass and maximize the production of pigments.

This study presents the optimization of fermentation conditions at different initial glucose concentrations and different C:N ratio. The optimum conditions were selected and the effect of ammonium nitrate addition in the biopigment chemical profile was determined. The pigment chemical profile was analyzed using NMR as also Q-TOF/MS, and the produced pigments were evaluated for pH stability, antioxidant activity and cytotoxicity in DLD1 cells.

## 2. Materials and Methods

### 2.1. Strains and Pre-Culture Media

Pigment production has been evaluated with the wild-type fungal strain *Penicillium purpurogenum* CBS 113139 that was purchased from the Westerdijk Fungal Biodiversity Institute–KNAW. Preparation of inoculum was carried out in 50 mL potato dextrose agar solid media in 250 mL Erlenmeyer flasks without agitation, at 30 °C for approximately 6 days, until sporulation. After sporulation, shake flasks were stored at 4 °C for two weeks maximum. Long-term storage of fungal spores was achieved in cryovials containing 50% (*v*/*v*) concentrated spore suspension in between 0.01% and 50% (*v*/*v*) pure glycerol, at −80 °C.

### 2.2. Shake Flask Fermentations

Fermentations for biocolorant production were carried out in 500 mL shake flasks with baffles, with a working volume of 100 mL. Effect of initial sugar concentration was evaluated at 30, 60 and 90 g/L glucose supplemented with 2 g/L yeast extract and 50 mM sodium citrate buffer, pH 5. The effect of C:N ratio was evaluated at different glucose to yeast extract concentrations. Glucose was 30 g/L and yeast extract concentrations of 2, 3, 4, 6, 12, 24 and 48 g/L of yeast extract corresponding to C:N ratios of 55:1, 36:1, 27:1, 18:1, 9:1, 5:1 and 2:1, respectively. The combination of the sugar and yeast extract concentrations were selected in order to evaluate a wide range of yeast extract addition in the fermentation medium. Sugar solution, yeast extract solution and citrate buffer solution were sterilized separately to avoid Maillard reactions, at 121 °C for 20 min. Moreover, sugar solution was 200 g/L in order to avoid caramelization of sugars in the autoclave. Inoculum size was 1×10^7^ spores/mL. Agitation and temperature were controlled at 180 rpm and 30 °C in a rotary shaker incubator.

### 2.3. Pigment Production at Optimum Conditions

The optimum conditions from the shake flask fermentations were selected for the production of a big batch of water-soluble pigments for further analysis. The fermentations were carried out in 2 L shake flasks with baffles, with 400 mL fermentation working volume. Initial glucose concentration was 30 g/L, and yeast extract was 3 g/L corresponding to a C:N ratio of 36:1. The pigments namely “Pigment Complex A” and “Pigment Complex B” were further evaluated for pH stability, their composition was analyzed and their cytotoxicity was evaluated. Pigment Complex A derived from a fermentation medium that contained 30 g/L of glucose, 3 g/L of yeast extract and 50 mM sodium citrate buffer, pH 5. Pigment Complex B derived from a fermentation medium that contained 30 g/L of glucose, 3 g/L of yeast extract, 3 g/L of ammonium nitrate, and 50 mM sodium citrate buffer, pH 5.

### 2.4. pH Stability

The stability of the pigments was evaluated at different pH values with a range from 1.1 to 11.5 for Pigment Complex A and a range of 0.7 to 11.3 for Pigment Complex B. The produced pigments were extracted with ethanol at pH 2. The pigments were freeze dried and were further solubilized in 50 mM sodium citrate buffer to provide buffering capacity and protect the pigments from extreme pH change. Different pH values were adjusted with the addition of 0.1 M of NaOH or 0.1 HCl. The absorbance of the pigments was measured at 400 nm, 470 nm and 500 nm using a 96-well microplate reader spectrophotometer (Infinite Pro 200, Tecan). All the samples were diluted to measure the absorbance at the linear area of the spectrophotometer (0.1–3 AU). The color of the samples was also measured using a colorimetry with output of values L*, a* and b*. The Lab model is an international standard for color measurement developed by the commission Internationale de l’éclairage (CIE) in 1976. The model consists of three values. The L* value, ranging from 0 to 100, which measures the luminance or lightness and the chromatic values a* and b*, ranging from −120 to +120, scaling the color from green to red and blue to yellow, respectively [10]. These data were used to calculate C and H according to the following equations:

(1) $$C^{*}=\sqrt{{\left(a^{*}\right)}^{2}+{\left(b^{*}\;\right)}^{2}}$$

(2) $$H^{*}=\tan^{-1\;}\frac{b^{*}}{a^{*}}$$

### 2.5. Analytical Methods

The growth of the microorganisms was determined via measurements of biomass through drying of the sediment in each sample. The sugars and organic acids were determined using a Shimadzu HPLC system with a Shimadzu RI detector and an Aminex HPX-87H (7.8 × 300 mm) column. The temperature of the column was 70 °C, and the mobile phase was a 10 mM H_2_SO_4_ aqueous solution with 0.6 mL/min flow rate. Consumption of free amino nitrogen (FAN) during fermentation was determined according to the ninhydrin colorimetric method promulgated in the European Brewery Convention [11]. Yellow, orange and red pigments were determined by measuring the absorbance in different wavelengths, 400 nm, 470 nm and 500 nm, respectively [6].

### 2.6. Purification of Pigments

Samples were centrifuged (10,000× *g*, 10 min, 4 °C), and supernatant was isolated and lyophilized. The obtained residue was dissolved in acetonitrile with 0.1% trifluoroacetic acid (TFA) and H_2_O with 0.1% TFA, and then it was filtered through CHROMAFIL syringe filters, PP/RC-20/15 MS, 0.20 um, 15 mm diameter and purified using high-pressure liquid chromatography (HPLC, Dionex Ultimate 3000). Gradients were formed with two solvents, A and B. Solvent A was H_2_O with 0.1% TFA; solvent B was acetonitrile with 0.1% TFA. A linear gradient was performed from 30% B to 90% B for 20 min. The flow rate was 5 mL/min. ^1^H-NMR and LC NMR spectra were recorded on a Bruker AV-500 spectrometer equipped with a TXI cryoprobe (Bruker BioSpin, Rheinstetten, Germany). All 1D (^1^H,) and 2D NMR (HSQC, HMBC) measurements were performed using standard Bruker pulse sequences. For the NMR measurements, the pigments were dissolved in DMSO-d6.

### 2.7. High-Resolution Mass Spectrometry (HRMS) Analysis

The high-resolution mass spectrometry (HRMS) analysis of the extracts was carried out on an Impact II QToF HR mass spectrometer (Bruker Daltonik GmbH, Bremen, Germany) coupled to an Elute UHPLC system (Bruker Daltonik GmbH, Germany). The data acquisition was performed using Compass software (Bruker, Germany), while the data were processed by Metaboscape 5.0 software (Bruker, Germany). The lyophilized extracts were dissolved in 50% acetonitrile (LC-MS grade, Thermo Fisher, Pittsburgh, PA, USA) in water (LC-MS grade, Thermo Fisher, PA, USA) to achieve a concentration of about 0.2 mg/mL. The following chromatography program was applied: 0.1% formic acid in water as solvent A and 0.1% formic acid in acetonitrile as solvent B, with gradients as follows: 1% B for 2 min, 1% to 99% B in 15 min and 100% B for 3 min, using a flow rate of 0.25 mL min^−1^. The separation was carried out on a Bruker Intensity Solo 2 C18, 2.1 × 100 mm RP column at a constant temperature of 35 °C. An injection volume of 5 µL was used for the analysis. The analysis was performed in positive and negative ESI modes sequentially. The ESI source was operated at a nebulizer pressure of 2.0 bar, and the dry gas rate was set to 8.0 L min^−1^ at a temperature of 200 °C. The MS/MS spectra were recorded in Auto MS/MS mode, resulting in collision energy stepping from 20 to 50 eV.

### 2.8. NMR Spectroscopy

^1^H-NMR and LC NMR spectra were recorded on a Bruker AV-500 spectrometer equipped with a TXI cryoprobe (Bruker BioSpin, Rheinstetten, Germany). All 1D (^1^H,) and 2D NMR (HSQC, HMBC) measurements were performed using standard Bruker pulse sequences. For the NMR measurements, the pigments were dissolved in DMSO-d6.

### 2.9. Antioxidant Capacity

To evaluate the free radical (antiradical) scavenging activity, the samples, Pigment complex A and B at a concentration of 200μg/mL, were allowed to react with a stable free radical, 2.2-diphenyl-2-picryl hydrazyl free radical (DPPH). The method was based on the method developed by Firuzi et al. with slight modifications [12]. The samples were diluted in MeOH at a concentration of 1 mg/mL. 200 μg/mL of each sample were mixed with methanolic solution DPPH at a concentration of 0.3 mM. The mixture was incubated in a dark place at 25 °C for 30 min. The reduction of the color of the DPPH radical, which is a sign of the antioxidant capacity of the tested signal, was measured at 517 nm. Ascorbic acid was used as positive control. The % scavenging capacity of the samples was determined through the following equation:

% (scavenging capacity) = 100 − [(A_sample_ − A_blank_) × 100/A_control DPPH_],

where A_sample_ = absorbance of the sample, A_blank_ = absorbance of the blank of each sample and A_control DPPH_ = absorbance of the DPPH solution at 517 nm. The samples were tested in duplicates.

### 2.10. Cytotoxicity Assay

The DLD1 colon cancer cell line was cultured in McCoy’s 5A medium (Hyclone) supplemented with 10% fetal bovine serum (Gibco ^®^, Thermo Fisher, Pittsburgh, PA, USA), 100 U/mL penicillin and 100 μg/mL streptomycin (Gibco ^®^, Thermo Fisher, Pittsburgh, PA, USA). The viability of DLD1 cells in the presence of Pigment Complex A and B was estimated through the MTT (3-[4,5-dimethylthiazol-2-yl]-2,5 diphenyl tetrazolium bromide) assay. Specifically, 3000 cells/well were seeded in 96-well plates. The cells were maintained at 37 °C in a humidified atmosphere of 5% CO_2_. The next day, stock solutions of Pigment Complex A and B were prepared in DMSO and were further diluted in McCoy’s 5A medium in order to achieve final concentrations of 5–500 μg/mL for Pigment Complex B and 5–300 μg/mL for Pigment Complex A, respectively. Pigment Complex A was less soluble in DMSO and thus concentrations up to 300 μg/mL were applied. The DMSO content did not exceed 0.5% of the final volume in each well to restrict its cytotoxic effect. The plates were incubated at 37 °C in a humidified atmosphere (5% CO_2_) for 72 h. Then, 10 μL of MTT solution (5 mg/mL in phosphate buffered saline (PBS)) were added in each well and incubated for 4 h. The medium was removed and 100 μL of DMSO were added in each well. The absorbance of the samples was measured via an Elisa plate reader (Awareness Technology Inc.) at 540 nm while a differential filter was set to 630 nm. Each concentration was applied in triplicates. Statistical analysis was carried out using GraphPad Prism 8. The effect of Pigment Complex A and B were compared to the DMSO treated cells using one-way ANOVA and Dunnett’s test. The *p*-value was considered at 5% level of significance.

## 3. Results and Discussion

### 3.1. Optimization of Initial Glucose Concentration and Carbon to Nitrogen Ratio

**Figure 1:**
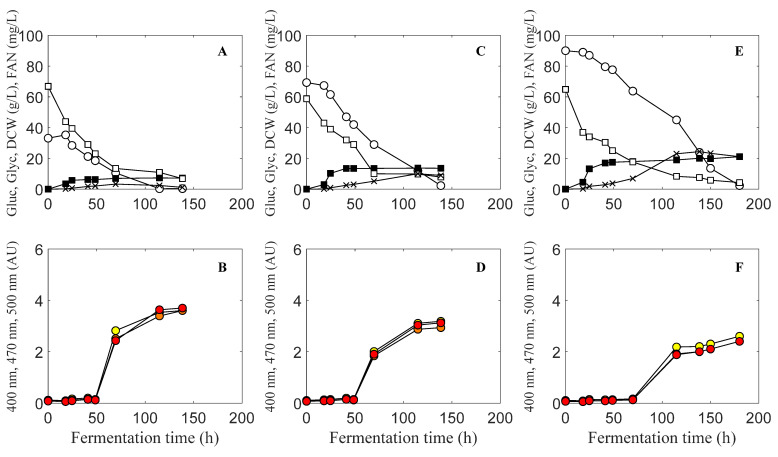
Glucose and free amino nitrogen (FAN) consumption and fermentation product accumulation at different initial sugar concentrations of 30 g/L (**A**,**B**), 60 g/L (**C**,**D**) and 90 g/L (**E**,**F**). Glucose (o). FAN (□). DCW (■). Glycerol (×). Absorbance at 400 nm (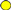). Absorbance at 470 nm (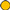). Absorbance at 500 nm (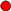).

The effect of initial glucose concentration on the production of biobased colorants by *Penicillium purpurogenum* was investigated at 30 g/L, 60 g/L and 90 g/L of commercial glucose with around 70 mg/L of initial free amino nitrogen concentration in all cases (Figure 1). Production of biocolorants started at around 50 h of fermentation after the depletion of free amino nitrogen (FAN). Biomass production stopped when FAN was depleted since biocolorants are secondary metabolites. Maximum biomass was 7.3 g/L, 13.7 g/L and 22.1 g/L for 30 g/L, 60 g/L and 90 g/L of initial glucose, respectively. Biocolorant production at 30 g/L of initial sugar concentration was 3.6 AU for 400 nm, 3.6 AU for 470 nm and 3.7 AU for 500 nm. At 60 g/L, initial sugar concentration biocolorant production was 3.2 AU for 400 nm, 2.9 AU for 470 nm and 3.1 AU for 500 nm, and at 90 g/L of initial glucose concentration, biocolorant production was 2.6 AU for 400 nm, 2.4 AU for 470 nm and 2.4 AU for 500 nm. Higher initial glucose concentrations resulted in glycerol production and may be associated with its osmoprotectant role towards high sugar concentration [13]. Specifically, final glycerol concentration was 1.2 g/L at 30 g/L initial glucose concentration, 8.9 g/L at 60 g/L initial glucose concentration and 27.1 g/L at 90 g/L initial glucose concentration. As it can be observed in Figure 1, higher initial sugar concentrations resulted in higher glycerol production and lower biocolorant production. Production of glycerol as a response to hyperosmotic fermentation conditions is known to occur in all microorganisms and specifically in yeast [14]. In our case, production of glycerol competed for carbon source consumption, resulting in lower pigment production at high initial glucose concentration, since a decrease in pigment production was observed at higher initial sugar concentrations. Final colorant absorbance (AU) decreased with increasing initial sugar concentrations at 400, 470 and 500 nm (Figure 1).

**Figure 2:**
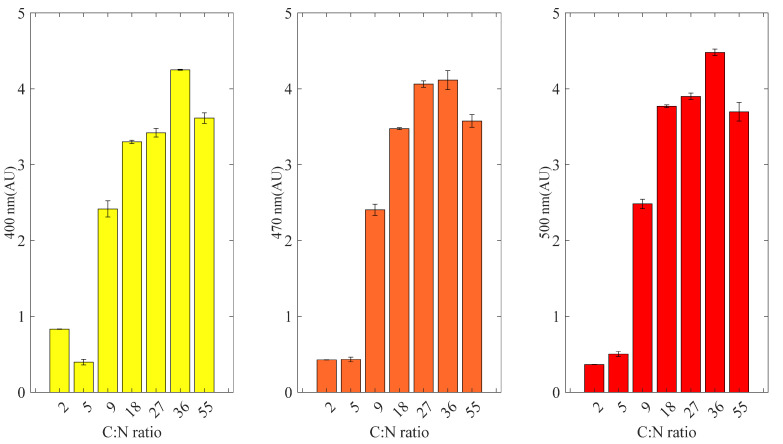
Final pigment production in fermentations with *Penicillium purpurogenum* at different carbon to nitrogen ratios (C:N) of 55:1, 36:1, 27:1, 18:1, 9:1, 5:1 and 2:1. Absorbance at 400 nm (yellow bars). Absorbance at 470 nm (orange bars). Absorbance at 500 nm (red bars).

The effect of carbon to nitrogen ratio was evaluated using commercial glucose as carbon source at initial concentration of around 60 g/L and different yeast extract concentrations (2, 3, 4, 6, 12, 24 and 48 g/L of yeast extract corresponding to C:N ratios of 55:1, 36:1, 27:1, 18:1, 9:1, 5:1 and 2:1, respectively). Colorant production was significantly low at C:N ratios lower than 9:1 resulting in maximum absorbance units lower than 1AU for all measured spectra (400 nm, 470 nm and 500 nm). Absorbance of color increased with increasing C:N ratio (Figure 2). Significant increase of biobased pigments was observed at C:N ratio of 36 and 55. Specifically, at 36:1 the biopigments reached 3.6 AU for 400 nm, 3.6 AU for 470 nm and 3.7 AU for 500 nm. At 55:1 the biopigments reached 4.3 AU for 400 nm, 4.1 AU for 470 nm and 4.5 AU for 500 nm (Figure 2). As a secondary metabolite, biobased colorants are produced during the stationary phase of biomass growth. Therefore, the appropriate C:N ratio that will offer sufficient carbon for both biomass and biobased colorants has to be selected in order to achieve high colorant production in batch fermentations. The optimum C:N ratio 55:1 was selected in order to produce biocolorants with supplementation of 3 g/L ammonium nitrate Pigment Complex B. Final absorbance of pigment production in Pigment Complex B was 4.1 AU for 400 nm, 4.5 AU for 470 nm and 4.9 AU for 500 nm (Table S1).

Fermentation conditions and medium composition can affect the type of pigments, the intensity of the color and the stability of the color that is produced by the fungal strains. Environmental factors such as exposure to light, fermentation pH and temperature, oxygen availability, carbon to nitrogen ratio and the presence of minerals will affect the production of the pigments [5,15]. The effect of initial glucose concentration and different C:N ratio was evaluated in *Monascus ruber*, a fungal strain that produces similar type of pigments with *Penicillium purpurogenum* [16]. The red pigments were proved to be growth associated and initial sugar concentration and C:N ratio affected both growth and pigment production. Fermentations at initial glucose concentrations of 5–25 g/L showed that the maximum production of pigments was 24.7 AU per g of biomass DCW, which was observed when initial glucose concentration was 10 g/L [16]. Red pigment production was also evaluated in *Monascus ruber* fermentations at different initial C:N ratios resulting in around 25 AU per g of biomass DCW at 9:1 C:N mol ratio [16]. Carbon to nitrogen ratio was also evaluated in submerged cultivation of medicinal mushroom *Cordyceps militaris* for the production of cordycepin, with the highest production of 345 mg/L and a productivity of 19.2 mg/L/d at 42 g/L of initial glucose and 15.8 g/L peptone [17].

### 3.2. pH Stability

**Figure 3:**
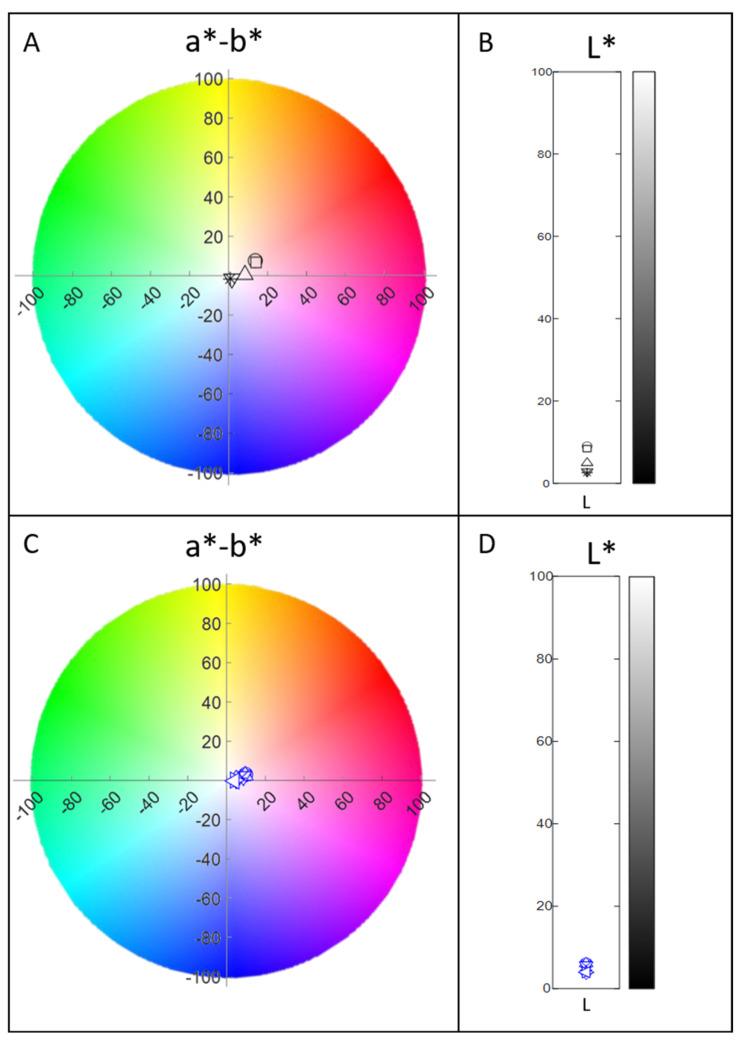
CIELAB graph presenting a*-b* and L* values given by the colorimetry at different pH values of extracted pigments from Pigment Complex A (**A**,**B**) and Pigment Complex B (**C**,**D**).

The stability of biocolorants was evaluated at different pH values and the absorbance of the samples was measured at 400, 470 and 500 nm using a spectrophotometer (Figure S1) and were also evaluated in a colorimetry (Figure 3 and Table S2). The color was relatively stable at a pH range from 4.3 to 10.5 with average AU of 7 at 400 nm, 6.8 at 470 nm and 7.8 at 500 nm. At pH values of 1.1 to 2.4, the AU of the color decreased at decreasing pH leading to a significant reduction at pH 1.1 where AU was 3.9 at 400 nm, 3.6 at 470 nm and 3.7 at 500 nm. Decreased color absorbance was also observed at increasing pH values (higher than 10.5) with significant reduction of absorbance at pH 11.5 with AU of 6.7 at 400 nm, 6.3 at 470 nm and 5.9 at 500 nm. The pigments produced with the addition of ammonium nitrate supplements (Pigment Complex B) resulted in good stability at a higher pH range (Figure S1). The absorbance was relatively stable at pH values from 2.6 up to 10.3 with average AU of 6.6 at 400 nm, 9.3 at 470 nm and 11.4 at 500 nm. Significant loss of absorbance was observed at pH values lower than 2.6. A decrease in color intensity was also observed at pH values higher than 10.3. As shown in Figure 3, pH values of 2.4 and 2.9 for Pigment Complex A, resulted in lower L values corresponding to lighter color while the a–b difference showed slight redness compared to the rest pH values. The colorimetry of Pigment Complex B was somewhat more stable than Pigment Complex A.

### 3.3. HR-MS and NMR Analysis of Biopigments

**Figure 4:**
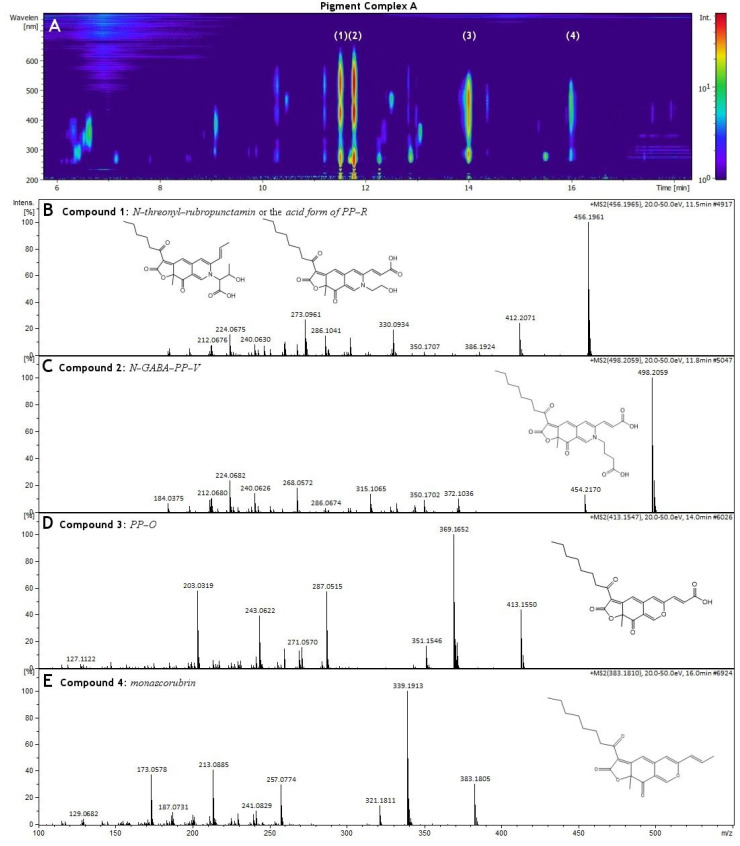
(**A**) Illustration of different compounds (Compound **1**–**4**) that were detected using UHPLC-DAD chromatography of Pigment Complex A. (**B**) ESI^+^-MS fragment of Compound **1** (*N-threonyl-rubropunctamin* or the *acid form of PP-R (purple-red pigments),* (**C**) ESI^+^-MS fragment of Compound **2** (*N-GABA-PP-V* or *6-[(Z)-2-Carboxyvinyl]-N-GABA-PP-V),* (**D**) ESI^+^-MS fragment of Compound **3** (*PP-O* or *(10Z)-12-carboxylmonascorubrin*), (**E**) ESI^+^-MS fragment of Compound **4** (*monascorubrin)*.

The pigments produced in Pigment Complex A were first analyzed by UV/Vis diode array chromatography. Using the same chromatographic conditions, we proceeded to the determination of these compounds using HR-MS. For the identification of the compounds, we searched the current literature for the known metabolites of *Penicillium purpurogenum* [18,19,20], collected all the reported compounds and created an in-house library containing their chemical structures. We used this library in conjunction with Bruker’s MetaboBASE Plant Library to annotate the compounds being reported in Table S3. The chromatographic analysis of Pigment Complex A illustrated four major pigments (Compounds **1**–**4**) in different retention times (Figure 4A). The calculated molecular mass of Compound **1** at a retention time of 11.5 min was determined as 455.194, with the proposed chemical formula C_25_H_29_NO_7_ and a mass error of 0.139 ppm. This compound was identified as either *N-threonyl-rubropunctamin* or the *acid form of PP-R (purple-red pigments)* (Figure 4B). However, since threonine was not detected in Pigment Complex A, which is necessary for the synthesis of *N-threonyl-rubropunctamin* [18], the *acid form of PP-R* is the most likely candidate. The calculated molecular mass of Compound **2** at a retention time of 11.8 min was determined as 497.205, the proposed chemical formula C_27_H_31_NO_8_ and a mass error of 0.617 ppm. It was identified as the polyketide azaphilone violet pigment *N-GABA-PP-V* or *6-[(Z)-2-Carboxyvinyl]-N-GABA-PP-V,* a homologue of the PP-V pigment (Figure 4C). The calculated molecular mass of Compound **3** at a retention time of 14.0 min was determined as 412.122, with the proposed chemical formula C_23_H_24_O_7_ and a mass error of only 0.247 ppm. This pigment was identified as the red-orange pigment *PP-O* or *(10Z)-12-carboxylmonascorubrin*. (Figure 4D). The calculated molecular mass of Compound **4** at a retention time of 16.0 min was determined as 382.178, the proposed chemical formula C_23_H_26_O_5_ and a mass error of 1 ppm. This pigment was identified as *monascorubrin* (Figure 4E).

**Figure 5:**
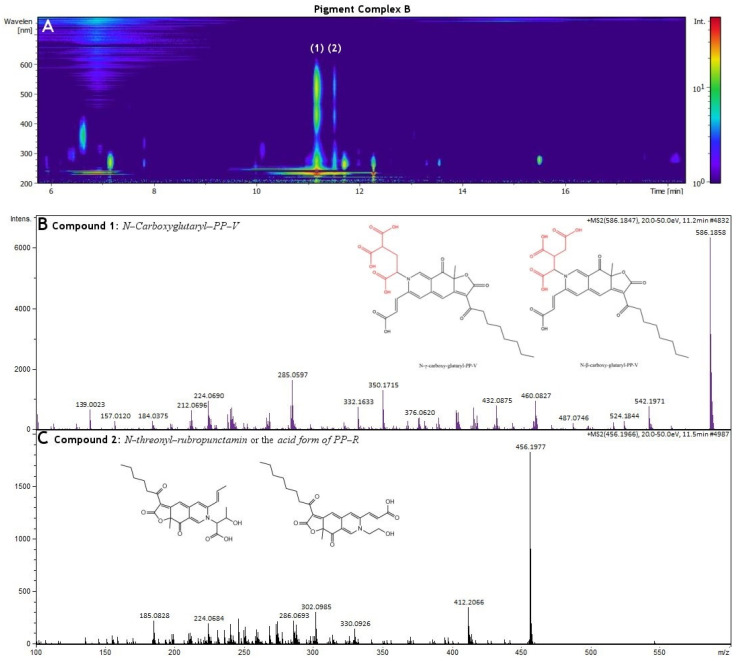
(**A**) Illustration of different compounds (Compound **1**–**2**) that were detected using UHPLC-DAD chromatography of Pigment Complex B. (**B**) ESI^+^-MS fragment of Compound **1** (*N-carboxyglutaryl-PP-V),* (**C**) ESI^+^-MS fragment of Compound **2** (*N-threonyl-rubropunctamin* or the *acid form of PP-R*).

The same procedure was followed for the pigment produced in the Pigment Complex B. It was initially analyzed by UV/Vis diode array chromatography. The chromatogram illustrated the detection of two different major compounds (Compound **1** and Compound **2**), at retention time of 11.2 min and 11.5 min, respectively. The 3D diode array signal is presented in Figure 5A. Sequentially, we proceeded to the chemical profile analysis of Pigment Complex B using HR-MS, and the list of annotated compounds is reported in supplementary materials. From this analysis, we identified a higher abundance pigment at the retention time of 11.2 min with a calculated molecular mass of 585.162, corresponding to the formula C_29_H_31_NO_12_ and a mass error of only 0.863 ppm. Interestingly, this structure has never been formerly reported in the existing literature. It shared three major fragments with N-GABA-PP-V at 410.161, 351.148 and 366.171. Its overall fragmentation is also very similar with N-GABA-PP-V, which indicates that it is a polyketide azaphilone pigment with the same carboxyl-monascorubramine base structure of PP-V. It contains two additional carboxylic groups with respect to N-GABA-PP-V (C_27_H_31_NO_8_, Figure 5), proposing a structure of N-C_6_H_6_O_6_-PPV. To identify this compound, we performed in silico fragmentation evaluations of any possible compounds matching the proposed formula. This leads to the conclusion that this new polyketide azaphilone pigment is the *N-carboxyglutaryl-PP-V* either in *N-beta-carboxyglutaryl-PP-V* or *N-gamma-carboxyglutaryl-PP-V* form. The determination of a high abundance of gamma-carboxyglutamic acid (GLA) residue in the extract Pigment Complex B agrees with our proposed structure for the isolated Compound **1** that could contain in its scaffold GLA (Table S3), since GLA can be used by *Penicillium purpurogenum* for the synthesis of the relevant biopigment [18]. Thus, the most likely candidate biopigment is *N-gamma-carboxyglutaryl-PP-V* form (N-GLA-PP-V). The gamma-carboxyglutamic acid was not detected in Pigment Complex A that also explains the lack of this novel pigment in that pigment complex.

A second pigment, Compound **2**, at a much lower amount was also identified at a retention time of 11.5 min. The calculated molecular mass of Compound **2** was determined as 455.194, with a mass error of 0.139 ppm, and the proposed chemical formula C_25_H_29_NO_7_. This compound was identified as either *N-threonyl-rubropunctamin* or the *Acid form of PP-R (purple-red pigments)* (Figure 5C), the same pigment that was detected in Pigment Complex A. However, since threonine was not detected in Pigment Complex B, which is necessary for the synthesis of *N-threonyl-rubropunctamin* [18], the *acid form of PP-R* is the most likely candidate. In Pigment Complex B, traces of *PP-O* and *monascorubrin* were also detected.

To further analyze the predominant pigment in Pigment Complex B, the pigment extract was purified with HPLC as described in the Materials and Methods section (Figure S3). A peak with a retention time 11.3 min was isolated resulting in a dark red powder after lyophilization. ^1^H-NMR spectroscopy indicated that the isolated compound contains four carboxylic acid groups at 12.3 ppm. Table S4 shows the values of ^1^H spectral data of the isolated pigment. The protons that correspond to C22 (Figure S2) are found at 2.65 ppm. In the case of the *N-beta-carboxyglutaryl-PP-V*, the protons that correspond to C23 (Figure S2) would be expected at higher chemical shifts due to the α-position regarding to the adjacent carboxylic acid. Thus, NMR analysis corroborates to the UHPLC-MS data indicating that the major pigment in Pigment Complex B is *N-GLA-PP-V*. By combining two different analytical techniques, HRMS spectrometry and ^1^H-NMR spectroscopy, we were able to identify with high accuracy the identified biopigments produced by *Penicillium purpurogenum* in the two complexes and determine the structure of the newly discovered biopigment.

### 3.4. Antioxidant Capacity (DPPH Test)

The determination of the antioxidant activity of the samples, through the method of the free radical DPPH, is a quick and widely used method. The decreasing of the intense purple color of the free radical DPPH is correlated with the degree of the antioxidant capacity of the tested sample [12]. The two samples, Pigment Complex A and B, were tested at a concentration of 200 μg/mL. The results showed that Pigment Complex A exhibited antioxidant activity with an EC50 value of 0.41 μg/mL, while Pigment Complex B exhibited antioxidant activity with an EC50 of 0.37 μg/mL. The ascorbic acid (control for DPPH) demonstrated an EC50 value of 5.01 μg/mL. Though the antioxidant activity of the samples is weak, Pigment Complex B was proved to have a little higher antioxidant activity at a concentration of 200 μg/mL than Pigment Complex A. Finally, the antioxidant activity of the newly isolated pigment, *N-GLA-PP-V*, showed similar antioxidant activity, with corresponding pigments that have been described in the literature [21] with an EC50 of 0.38 μg/mL.

### 3.5. Cytotoxicity Assay

**Figure 6:**
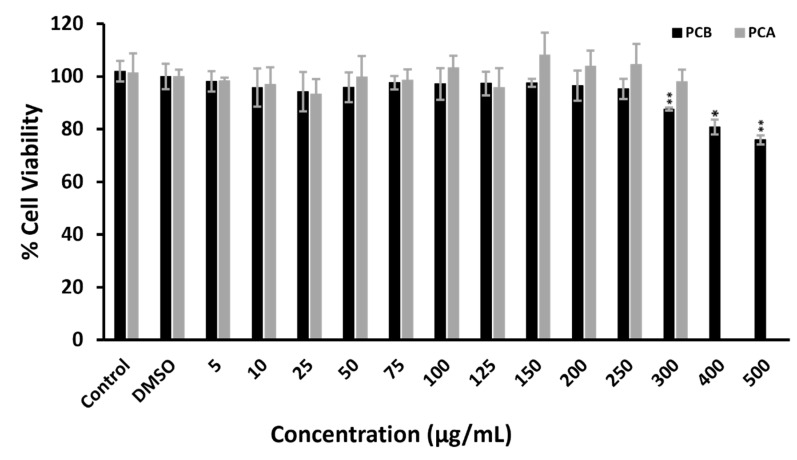
Cell viability of DLD1 cells under treatment with Pigment Complex A (PCA) and Pigment Complex B (PCB). Cells were treated with the indicated concentrations for 72 h. and the viability was estimated by the MTT assay. Control corresponds to the untreated cells and DMSO to the highest volume (0.5% *v*/*v*) of DMSO which was added to the cells. Statistical analysis was conducted with the Dunnett’s test. ** *p* < 0.01. * *p* < 0.1.

Biobased pigments are generally considered safe for humans compared to synthetic pigments. On the other hand, biobased pigments can exert significant bioactivity, such as anticancer [22,23], anti-inflammatory [24] and antidiabetic [25]. Herein, in order to explore the possible cytotoxic effect of the two pigment extracts in vitro, we deployed the DLD1 colon cancer, epithelial cell line. DLD1 cells are intestinal, epithelial cells and thus can serve as a model to determine the cytotoxicity of edible or orally administered substances. The viability of DLD1 cells in the presence of various concentrations of Pigment Complex B (5–500 μg/mL) and Pigment Complex A (5–300 μg/mL) for 72 h was assessed through the MTT assay. Interestingly, no cytotoxic effect was observed for Pigment Complex B at any of the indicated concentrations. Following a different pattern, Pigment Complex B exhibited no cytotoxicity up to 250 μg/mL; however, treatment at 300, 400 and 500 mg/mL slightly, but significantly, decreased the cell viability at 87.5 ± 0.5, 80.7 ± 2.8 and 75.9 ± 1.7, respectively (Figure 6).

## 4. Conclusions

This work focused on the production of biobased pigments via *Penicillium purpurogenum* fermentation. Optimization of initial sugar concentration and C:N ratio in *Penicillium purpurogenum* fermentations resulted in significant production of biobased colorants. Specifically, optimum initial sugar concentration was 30 g/L and resulted in 3.6–3.7 AU of pigments, while higher sugar concentrations shifted the metabolism towards glycerol production. Optimum C:N ratio was 36:1 in batch fermentation mode, corresponding to 30 g/L of initial sugars and 3 g/L of yeast extract, resulting in 4.1–4.5 AU for Pigment Complex A and 4.1–4.9 AU for Pigment Complex B (supplemented with ammonium nitrate). Pigment stability resulted in stable color at a pH range of 4.3–10.5 for Pigment Complex A and 2.3–10.3 for Pigment Complex B. A new pigment in Pigment Complex B, that has the carboxyl-monascorubramine base structure as PP-V (violet pigment), was named as *N-GLA-PP-V*. Antioxidant activity evaluation indicated that both extracts illustrate similar antioxidant capacity. Cytotoxicity assays in colon cancer epithelial cells indicated that both extracts are not cytotoxic up to a concentration of 250 μg/mL.

## Data Availability

Not applicable.

## Supplementary Materials

The following supporting information can be downloaded, Table S1: Fermentation efficiency of biobased pigment production by Penicillium purpurogenum at different initial glucose concentrations (30, 60 and 90 g/L) and different carbon to nitrogen ratio (C:N) of 55:1, 36:1, 27:1, 18:1, 9:1, 5:1 and 2:1. Table S2: Color coordinates for CIELAB color space of pigment stability. Table S3: List of annotated compounds in “Pigment Complex A” and “Pigment Complex B” using High Resolution Mass Spectrometry. Table S4: ^1^H NMR Assignments of isolated compound of “Pigment Complex B” sample. Figure S1: Stability of pigments produced by Penicillium purpurogenum at different pH values ranging from 1 to 12. Figure S2: Proposed structures of predominant compound of “Pigment Complex B”. Figure S3: HPLC chromatogram of Pigment Complex B. Figure S4: Structure and 1HNMR spectra of the isolated major pigment in Pigment Complex B sample.

## Sample Availability

Samples are available from the authors.
