# Supplementary material for: Chemical Profiling, Bioactivity Evaluation and the Discovery of a Novel Biopigment Produced by Penicillium purpurogenum CBS 113139
Source: Molecules. 2021 Dec 23;27(1):69. doi: 10.3390/molecules27010069 (PMC8746843; doi:10.3390/molecules27010069)
Supplement: Supplementary file 1 [file molecules-27-00069-s001.zip › molecules-1451488-supplementary.pdf]

Chemical profiling, bioactivity evaluation and the discovery of a novel biopigment produced by *Penicillium purpurogenum* CBS 113139

Antonis D. Tsiailanis[a,ⱶ], Chrysanthi Pateraki[b,ⱶ], Kyriazou Mary[b], Christos Chatzigiannis[a], Maria Chatziathanasiadou[a], Nikolaos Parisis[a,c], Ioanna Mandala[b], Andreas G. Tzakos[a,c,d,*], Apostolis Koutinas[b,*]

[a] Department of Chemistry, Section of Organic Chemistry and Biochemistry, University of Ioannina, Ioannina, Greece

[b] Department of Food Science and Human Nutrition, Agricultural University of Athens, Iera Odos 75, 118 55 Athens, Greece

[c] Department of Chemistry, Biomedical and Analytical Center (BAC), University of Ioannina, Ioannina, Greece

[d] University Research Center of Ioannina (URCI), Institute of Materials Science and Computing, Ioannina, Greece

ⱶ Equally contributed as first authors

* Equally contributed as corresponding authors, E-mails: akoutinas@aua.gr, atzakos@uoi.gr, Tel/Fax: +302105294729

Contents

Table S1 Fermentation efficiency of biobased pigment production by *Penicillium purpurogenum* at different initial glucose concentrations (30, 60 and 90 g/L) and different carbon to nitrogen ratio (C:N) of 55:1, 36:1, 27:1, 18:1, 9:1, 5:1 and 2:1. 3

Table S2 Color coordinates for CIELAB color space of pigment stability produced by *Penicillium purpurogenum* at different pH values ranging from 2.4-11.5 for Pigment Complex A and 1.8-12.3 for Pigment Complex B. Chroma (C*) was calculated according to Equation (1). Hue angle (H) was calculated according to Equation (2). L* indicates lightness read from 0 (black) to 100 (white). The positive a* value indicates the red color while the negative a* value represents the green color. Similarly positive and negative b* values indicate the yellow and the blue colors respectively. Chroma values denote the saturation or purity of color. Hue angle values represent the degree of redness, yellowness, greenness, and blueness; the maximum is at 0, 90, 180 and 270 degrees, respectively. 4

Table S3 List of annotated compounds in "Pigment Complex A" and "Pigment Complex B" using High Resolution Mass Spectrometry 5

Table S4. ^{1}H NMR Assignments of isolated compound of "Pigment Complex B" sample. The abbreviations are s for singlet, d for doublet, t for triplet, m for multiplet and nf stands for not found. 8

Figure S1 Stability of pigments produced by *Penicillium purpurogenum* at different pH values ranging from 1 to 12. A. Pigments from fermentation medium without ammonium nitrate. B. Pigments from fermentation medium with ammonium nitrate. Absorbance at 400 nm (●). Absorbance at 470 nm (●). Absorbance at 500 nm (●). 10

Figure S2 Proposed structures of predominant compound of "Pigment Complex B". 11

Figure S3 HPLC chromatogram of Pigment Complex B 11

FigS4. Structure and ^{1}HNMR spectra of the isolated major pigment in Pigment Complex B sample. 12

Table S1 Fermentation efficiency of biobased pigment production by *Penicillium purpurogenum* at different initial glucose concentrations (30, 60 and 90 g/L) and different carbon to nitrogen ratio (C:N) of 55:1, 36:1, 27:1, 18:1, 9:1, 5:1 and 2:1.

	Final fermentation time (h)	AU (400 nm)	AU (470 nm)	AU (500 nm)	μmax (h-1)	Xmax (g/L)	Y X/S	Specific productivity (AU/gDCW/h)	Glycerol (g/L)
				Initial glucose concentration					
30	138	3.6	3.6	3.7	1.73	7.3	0.22	0.0042	1.2
60	138	3.2	2.9	3.1	2.46	13.7	0.20	0.0017	8.94
90	180	2.6	2.4	2.4	2.68	21.1	0.24	0.0007	21.1
				C:N ratio					
2:1	72	0.83	0.43	0.37	3.21	29.14	0.39	0.0004	2.1
5:1	72	0.40	0.43	0.50	3.12	27.13	0.36	0.0002	3.23
9:1	72	2.4	2.4	2.5	3.05	25.00	0.34	0.0014	3.84
18:1	72	3.3	3.5	3.8	3.11	29.75	0.54	0.0016	4.59
27:1	122	3.4	4.1	3.9	2.77	20.25	0.27	0.0014	3.65
36:1	143	4.3	4.1	4.5	2.66	23.25	0.33	0.0013	4.13
55:1	138	3.61	3.58	3.70	2.46	13.70	0.20	0.0019	3.78

Table S2 Color coordinates for CIELAB color space of pigment stability produced by *Penicillium purpurogenum* at different pH values ranging from 2.4-11.5 for Pigment Complex A and 1.8-12.3 for Pigment Complex B. Chroma (C*) was calculated according to Equation (1). Hue angle (H) was calculated according to Equation (2). L* indicates lightness read from 0 (black) to 100 (white). The positive a* value indicates the red color while the negative a* value represents the green color. Similarly positive and negative b* values indicate the yellow and the blue colors respectively. Chroma values denote the saturation or purity of color. Hue angle values represent the degree of redness, yellowness, greenness, and blueness; the maximum is at 0, 90, 180 and 270 degrees, respectively.

Pigments	pH	L	a	b	C	h
Pigment Complex A (without ammonium nitrate)	2.4	9.08	13.69	7.59	15.65	29.00
	2.9	8.45	14.08	6.71	15.60	25.48
	4.1	4.96	8.38	0.44	8.39	3.01
	6.8	2.85	1.69	-1.59	2.32	316.75
	10.8	2.51	0.94	-1.57	1.83	300.91
	11.5	2.63	0.88	-1.75	1.96	296.70
Pigment Complex B (with ammonium nitrate)	1.8	6.30	9.84	3.03	10.30	17.12
	2.2	5.95	9.83	2.57	10.16	14.65
	2.7	6.1	9.59	2.48	9.91	14.50
	3.3	5.7	8.69	2.04	8.93	13.21
	4	5.48	8.04	1.7	8.22	11.94

	5.6	4.48	6.86	1.17	6.96	9.68
	8.5	3.76	5	0.44	5.02	5.03
	9.5	3.95	4.34	0.15	4.34	1.98
	12.3	4	4.04	-0.26	4.05	356.32

Table S3 List of annotated compounds in "Pigment Complex A" and "Pigment Complex B" using High Resolution Mass Spectrometry

Compound Name	Retention time (min)	Pigment Complex A (Intensity)	Pigment Complex B (Intensity)
13Z-Docosenamide	15.2	1607	8355
17.18-dehydro-clavulone I	14.6	4409	0
2-(2-Butoxyethoxy)ethanol	8.4	7381	7931
2-(2-Butoxyethoxy)ethyl acetate	10.8	2300	2541
2.2.4-Trimethyl-1.3-pentanediol diisobutyrate	16.3	2006	2432
2.3-Dimethylaniline	1.4	11841	12685
2.4.5-Trihydroxytoluene	6.4	2541	1817
2-[2-(2-Butoxyethoxy)ethoxy]ethanol	9.7	14003	15565
2-Hydroxy-2-methylbutanedioic acid	1.4	2690	3812
2-Isopropylmalic acid	6.3	13229	13070
2-Methyl-1-Pyrroline	3.6	8542	7976

2-Methylimidazole	20.1	6642	6729
3-Hexynoic acid	6.0	24896	38061
4-Methylcatechol	5.1	1279	1890
4-Nitrophenol	9.0	2124	1073
5-keto-n-caproic acid	6.0	1276	5314
6-Hydroxyhexan-6-olide	3.4	8242	14453
7-Methoxychromone	12.1	12096	14736
Azelaic acid	8.5	6869	7236
Betaine	1.2	80864	131672
beta-Sitostenone	20.2	2851	0
Byssochlamic Acid	13.6	769	8526
Carboxyibuprofen	9.1	23474	2382
Choline	1.1	6574	16160
Citric acid	1.4	622997	626418
Cyanoacetic acid	18.8	4327	4240
D-(+)-Malic acid	1.3	579	3758
D-Lysine	1.0	0	3764
Ethylmalonic acid	5.2	710	2250
Ethylparaben	13.3	3482	1895
**gamma-carboxyglutamic acid**	**1.2**	**0**	**249459**
Hexamethylphosphoramide	7.7	48225	229
Homogentisic acid	5.1	2094	3559
Hydroxyphenyllactic acid	6.0	3844	2955
L-Alloisoleucine	2.8	169	6702

L-Arabitol	1.2	12547	6235
L-Aspartic Acid	1.1	97	11078
Lauroyl diethanolamide	13.3	2381	1307
L-Carnitine	1.2	5216	15571
L-Glutamic acid	1.1	0	64558
L-Histidine	1.0	0	3418
L-Iditol	1.1	64994	70025
L-Phenylalanine	5.0	97	6173
L-Tryptophan	2.8	0	8258
L-Tyrosine	2.8	0	6537
Mesaconic acid	1.4	79558	84044
Mevaldate	1.2	10666	0
Monascorubrin	***16.0***	***83693***	***794***
N-(2-Phenylethyl)-acetamide	8.5	247	8122
N-Acetyltyramine	6.6	121	4669
N-Butylbenzenesulfonamide	11.6	5656	7499
N-threonyl-rubropunctamin *or* ***Acid form of PP-R***	***11.5***	***161531***	***5353***
N-Acetyl-L-lysine	1.4	0	9781
Phenylpyruvic acid	7.2	1309	3718
Pinolenic Acid	14.6	7957	0
PP-O	***14.0***	***97367***	***833***
N-GABA-PP-V	***11.8***	***200199***	***0***
Protocatechuic acid	6.8	1010	9867
Pyruvate	1.4	0	9454

Suberic acid	7.8	3408	3618
Triethylamine	1.4	206874	183735
N-carboxyglutaryl-PP-V	***11.2***	***0***	***52498***
Uridine	1.4	1333	17539

Table S4. ^{1}H NMR Assignments of isolated compound of "Pigment Complex B" sample. The abbreviations are s for singlet, d for doublet, t for triplet, m for multiplet and nf stands for not found.

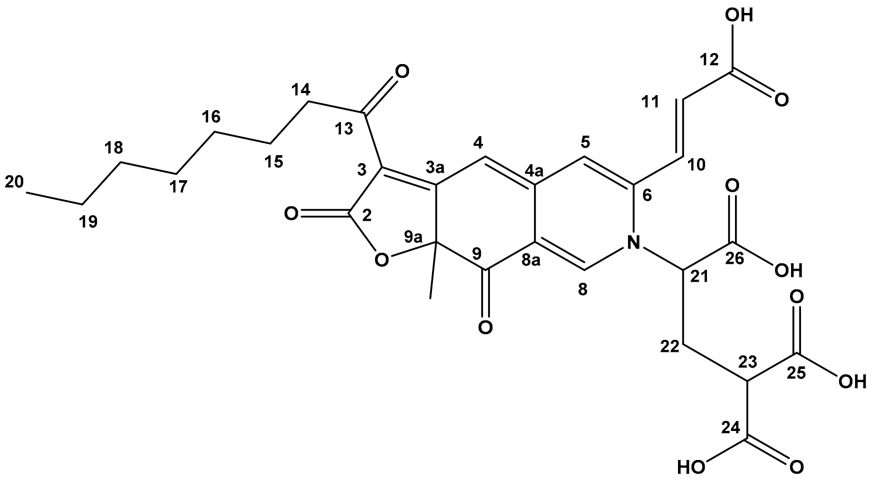

Atom No	δ_H	Multiplicity, J_c	Integral
2	-	-	-
3	-	-	-
3a	-	-	-
4	5.75	(s)	1H
4a	-	-	-
5	7.19	(s)	1H
6	-	-	-

8	8.27	(s)	1H
8a	-	-	-
9	-	-	-
9a	-	-	-
9a-CH_3	1.64	(s)	3H
10	6.71	(d), 8.047Hz	1H
11	7.03	(d), 8.072Hz	1H
12	-	-	-
13	-	-	-
14	2.65	(m)	2H
15	1.63	(m)	2H
16	1.63	(m)	2H
17	1.55	(m)	2H
18	0.9-0.95	(m)	2H
19	0.9-0.95	(m)	2H
20	0.89	(t), 7.23Hz	3H
21	nf	-	-
22	2.65	(m)	2H
23	nf	-	-
24	-	-	-
25	-	-	-
26	-	-	-

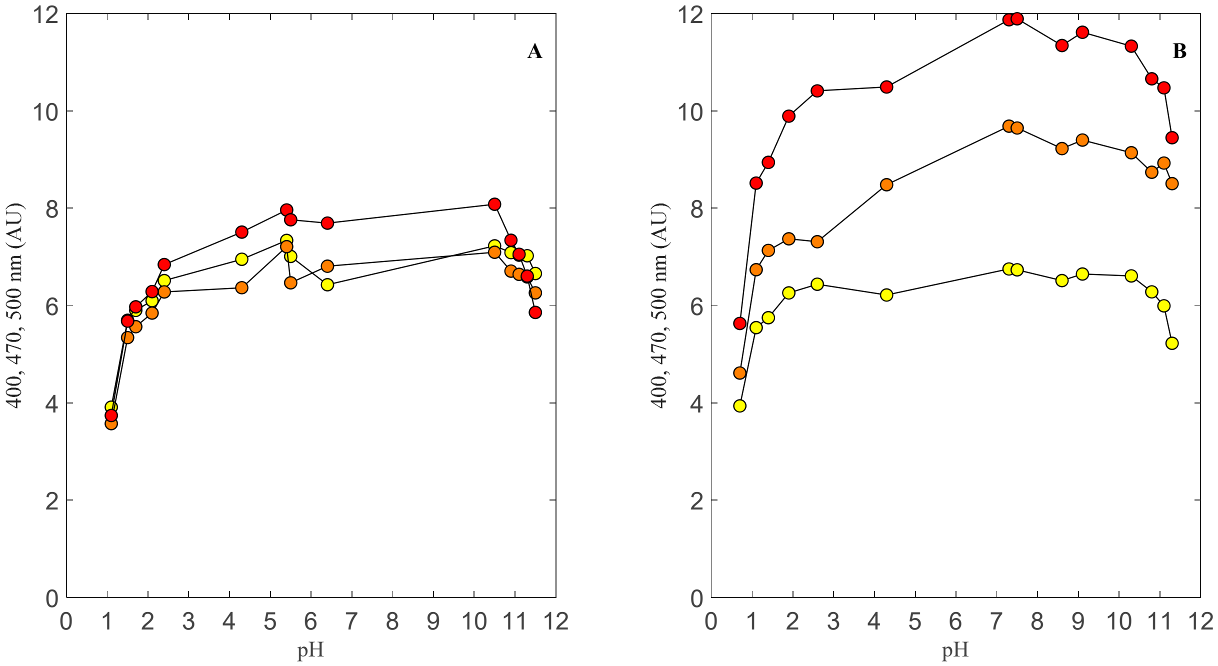

Figure S1 Stability of pigments produced by *Penicillium purpurogenum* at different pH values ranging from 1 to 12. A. Pigments from fermentation medium without ammonium nitrate. B. Pigments from fermentation medium with ammonium nitrate. Absorbance at 400 nm (●). Absorbance at 470 nm (●). Absorbance at 500 nm (●).

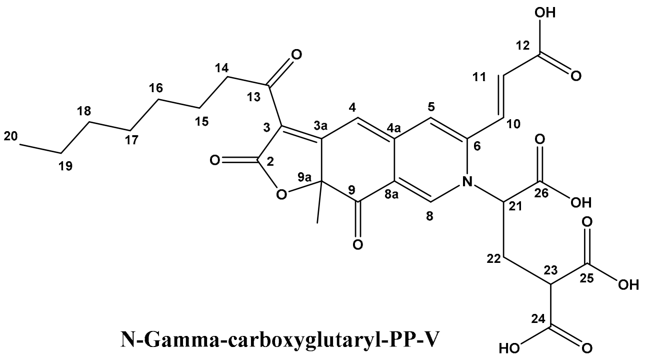

N-Gamma-carboxyglutaryl-PP-V

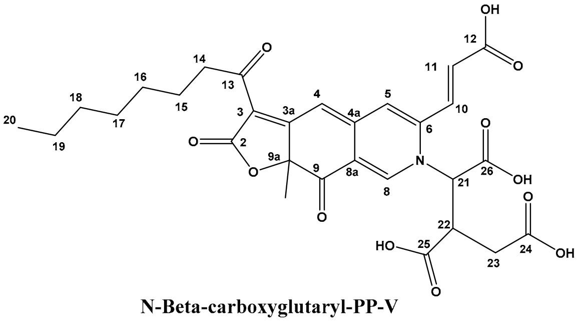

N-Beta-carboxyglutaryl-PP-V

Figure S2 Proposed structures of predominant compound of “Pigment Complex B”.

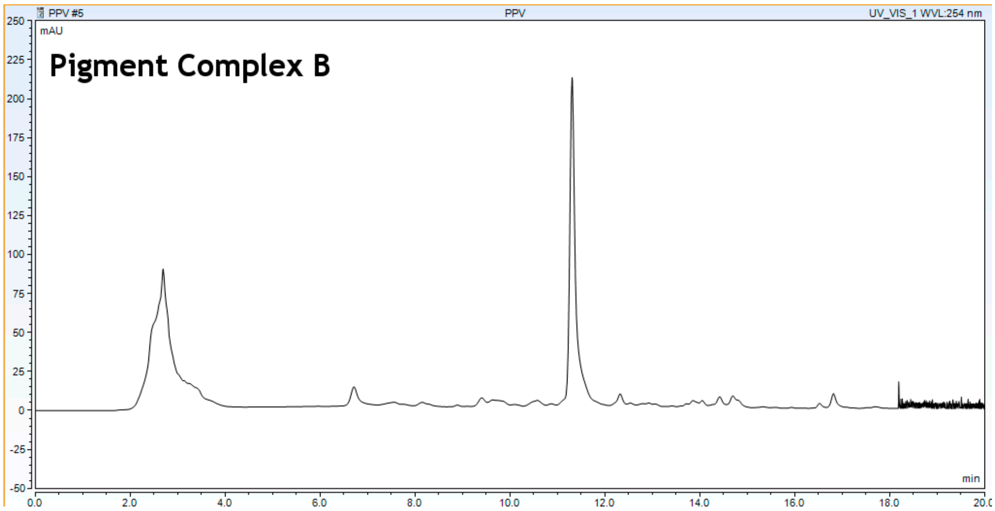

Figure S3 HPLC chromatogram of Pigment Complex B

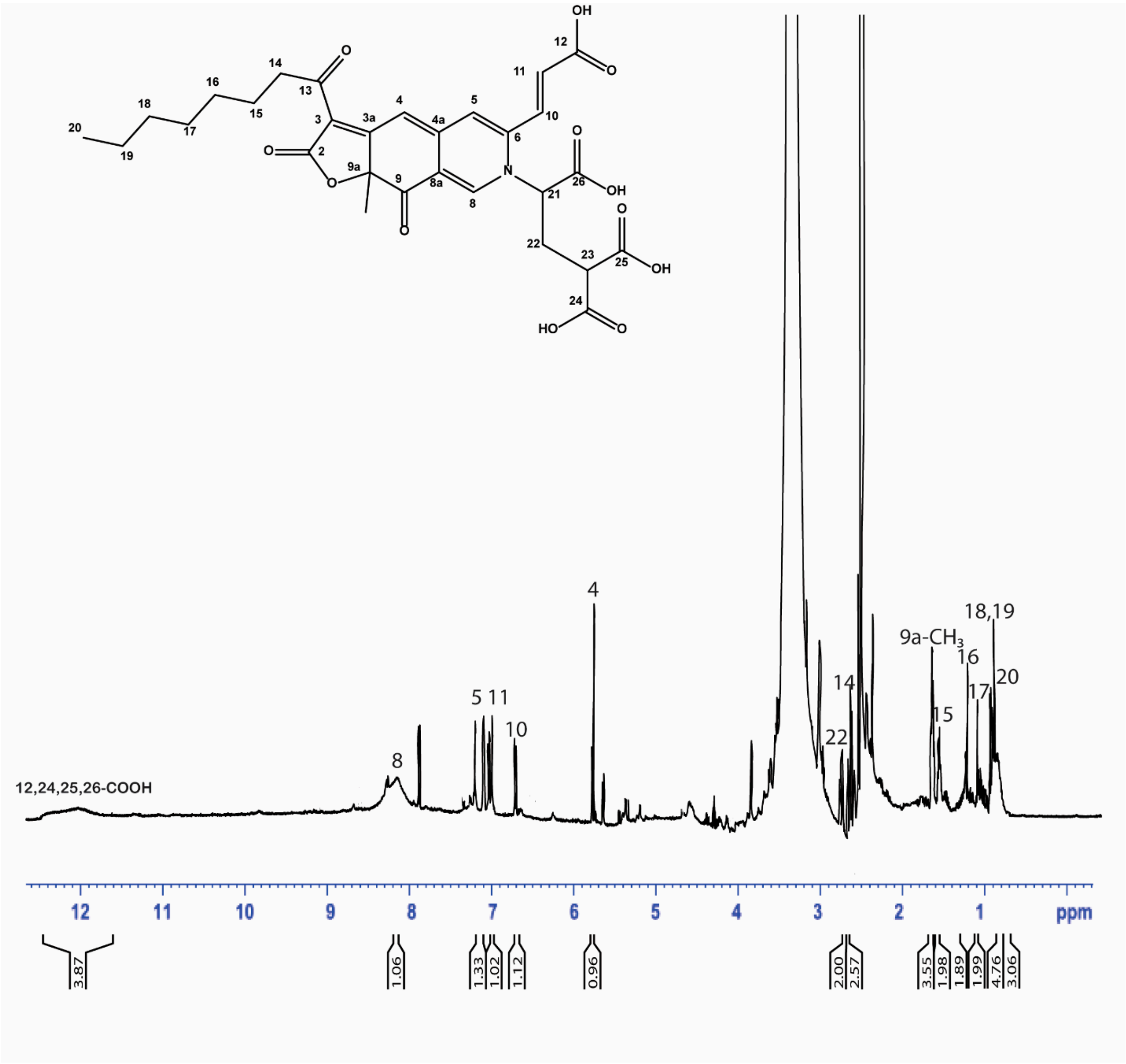

Figure S4. Structure and ^{1}HNMR spectra of the isolated major pigment in Pigment Complex B sample.
